# Evaluation of two health education interventions to improve the varicella vaccination: a randomized controlled trial from a province in the east China

**DOI:** 10.1186/s12889-018-5070-0

**Published:** 2018-01-16

**Authors:** Yu Hu, Qian Li, Yaping Chen

**Affiliations:** Department of Expanded Program on Immunization, Zhejiang Provincial Center for Disease Control and Prevention, No. 3399 Binsheng Road, Binjiang District, Hangzhou, People’s Republic of China

**Keywords:** Health education, Varicella vaccine, Randomized controlled trial, Effect, Evaluation

## Abstract

**Background:**

We evaluated the effect of two Elaboration Likelihood Model (ELM)-based health educational interventions on varicella vaccine (VarV) vaccination among pregnant women in a province in the east China.

**Methods:**

A prospective randomized controlled trial was conducted among 200 pregnant women with ≥12 gestation weeks to test two interventions, including a messaging video and a messaging booklet. The participants were randomly assigned into the control group, the video group or the booklet group. The VarV coverage at 12 and 24 months old was compared among the children of the three groups and relative risks (*RR*s) were calculated, by using the coverage of the control group as reference. The timeliness of VarV was also assessed. Furthermore, differences in the effects on the knowledge and attitude of VarV vaccination between the two interventions was evaluated.

**Results:**

The VarV coverage of their children by 24 months of age was 86.4%, 76.1% and 56.7% for the video group, the booklet group and the control group, respectively. The relative risks (*RR*s) for the coverage of VarV at 24 months of age were 4.8 (95% *CI*: 2.06–11.3) for the video group and 2.4 (95% *CI*: 1.2–5.1) for the booklet group. The means of delays were 57.3 days in the video group, 76.9 days in the booklet group, and 100.6 days in the control group. The proportion of women who intended to vaccinate their children with VarV was higher in the video group than the booklet group (93.9% vs. 82.1%, *p* < 0.05).

**Conclusions:**

Our findings indicated that perinatal health education through booklet or video could improve the coverage and schedule adherence for children’s VarV vaccination.

## Background

Varicella is a highly contagious disease caused by varicella-zoster virus primary infection. Varicella was a universal childhood disease before the era of varicella vaccine (VarV), which was developed in 1970s and is widely used by now [1]. The VarV has been approved for use in Zhejiang province since 1998, and Zhejiang provincial center for disease control and prevention (ZJCDC) published its initial recommendations in 2000, advising one dose of VarV to children at 12 months of age. The VarV can be immunized simultaneously with other vaccines on the same clinic day, at different anatomic sites. There is an exception that if the VarV is not immunized concurrently with other live attenuated vaccines (like measles containing vaccine), it should be immunized at least 28 days later. Despite this recommendation, childhood VarV vaccination in Zhejiang province remains suboptimal as the VarV vaccination has not been included in the routine immunization schedule. That means the VarV vaccination is voluntary and parents need to pay for their children’s vaccination with private fee. It is necessary to develop the evidence-based strategies for improving the coverage of VarV. Tailored messaging based on the elaborating likelihood model (ELM) framework has been successful in improving the coverage of human papillomavirus vaccine [2], and may be helpful in improving the childhood VarV coverage. The ELM describes two types of information processing including central route and peripheral route [3]. Central route is evoked when an individual has the motivation and ability to analyze a message, which tends to result in stronger behavioral changes. Peripheral route is evoked when simplistic messages or peripheral cues are used, which tends to result in less enduring behavioral changes. In the context of a more enduring behavioral change required for vaccination, health education interventions that utilize the central route may be more appropriate. However, it is unclear how the message of vaccination can be effectively delivered and very limited data exist on rigorously assessing its effects on the coverage of VarV.

This study aimed to: (1) evaluate the effects of the two different education interventions based on the ELM, with respect to the improvement in the coverage of VarV; (2) assess the timeliness of VarV vaccination by calculating the interval from the date of birth to the actual date of vaccination; (3) evaluate the difference in the effects on the knowledge and attitude of VarV vaccination between two interventions. This study might help to close the substantial evidence gap for rigorously evaluated health education interventions for increasing the acceptance and the coverage of VarV.

## Methods

### Study design and setting

This study was a prospective, randomized, controlled trial and the study population consisted of the pregnant women who were ≥12 gestational weeks in Changxing County, Zhejiang Province, East China. The total population of Changxing was 628,175 according to the census data of 2013 from Zhejiang provincial bureau of statistics. Four obstetric hospitals, which had the highest number of delivery in 2013, participated in this study as the enrollment sites. The selected hospitals served the population of pregnant women who were socio-economically diverse and were representative to the general population of pregnant woman in Changxing County.

### Sample size

The sample size was estimated using the formula as follows:$\mathrm{N}=\frac{{\left({z}_{\alpha}\sqrt{2\overline{p}\ \overline{q}}+{z}_{\beta}\sqrt{p_0{q}_0+{p}_1{q}_1}\right)}^2}{{\left({p}_1-{p}_0\right)}^2}$. The coverage of VarV in each intervention group was assumed as 60% (*p*_1_) while the coverage in the control group was assumed as 30% (*p*_0_). Therefore, 56 subjects in each treatment group was sufficient to detect a 30% difference in the coverage of VarV, with a power of 80% at a two-sided significance level of 0.05. Finally, 204 subjects in total or 17 subjects in each intervention group for every selected hospital were required with an attrition rate of 20%.

### Recruitment

Pregnant women with ≥12 gestational weeks were eligible for inclusion. They were approached by the trained midwives or physicians in the waiting rooms of the prenatal checkups at each hospital, from 1 Jan to 31 Mar, 2014. They were asked if they were willing to participate in an interview on the health education program on VarV. An informed consent was read to her and a written informed consent was obtained before every pregnant woman participated in our study. Demographic data including maternal age, maternal education level, maternal employment status, maternal immigration status, gestational week, number of children in the household were collected by a standard baseline questionnaire.

### Randomization and interventions

Randomization lists were produced separately for every four hospital by a non-study personnel. Enrolled women were assigned into the control group or one of the two intervention groups that were based on the ELM central processing route: (1) an affective messaging video or (2) a cognitive messaging booklet. The video was completed on a handheld electronic tablet device and both the two interventions were designed to take no more than 15 min, to enable participants to complete them while waiting for their appointments.

The video was tailored specifically to the pregnant women and showed a doctor providing the detailed information on VarV vaccination, the severity of varicella, the safety profile of VarV, and the current recommendation on VarV vaccination. The booklet provided the information through a question-answer format on the topic of the VarV vaccination, the disease burden of childhood varicella, the vaccine safety, and the current recommendation. The participants in the control group did not receive any educational instructions or materials. Study personnel recorded the time that participants spent on the interventions, and observed the women’s the attitude to the intervention activities. Study personnel assigned a score scaled from 1 to 5 for each participant in both two intervention groups (1 = very disengage, 2 = disengage, 3 = neither disengage nor engage, 4 = engage, 5 = very engage). Observation by study personnel was done in an unobtrusive manner to minimize the effect on participants’ experiences with the interventions. Those participants assigned into the two intervention groups were required to complete a post-intervention questionnaire, which asked them about the information learned, the confidence and comprehension on the information presented and the intention to receive VarV for their children.

The VarV vaccination status of the participants’ children in this study was extracted at 24 months of age from Zhejiang provincial immunization information system (ZJIIS). The function of ZJIIS was described previously elsewhere [4].

### Definition

In this study, we used two definitions of the coverage of VarV. The main reason for defining two criterion was that we want to evaluate not only the crude coverage of VarV, but also the timeliness of VarV vaccination. Specifically, the vaccination coverage was defined as the percentage of children who had received the VarV at the age of 24 months when the vaccination status was verified in ZJIIS, while the timeliness of VarV vaccination was defined as vaccination occurring before the end of the 12th month of age. The delay of VarV vaccination was defined as vaccination occurring within the time period the 13rd (366) to the 24th (732) months (days) of age. The non-vaccinated children at their 24 months of age were considered as missing values.

### Outcome

First, the coverage of VarV was compared among the three groups at 12 and 24 months of age. Additionally, we also compared the coverage of VarV at 24 month of age with the 4th dose of diphtheria-tetanus-pertussis combined vaccine (DTP4), which was one of the mandatory vaccines and scheduled at 18 months of age. The purpose for comparing the coverage of VarV and DTP4 at 24 months of age was that we want to evaluate the difference in the coverage between vaccine included in expanded immunization program and not. Second, the relative risks (*RR*s) for the coverage of VarV between the two intervention groups and the control group (reference) at different age were compared. The *RR*s between the coverage of VarV and DTP4 (reference) among the three randomization groups were also compared at 24 months of age. Third, we assessed the timeliness of VarV vaccination by calculating the interval, in days, from the date of birth to the actual date of vaccination. Fourth, the difference in the effects on the knowledge and attitude of VarV vaccination between the two interventions was evaluated by comparing the positive response rates between the two intervention groups. Fifth, we evaluated the engagement of the two intervention activities as additional findings.

### Data analysis

We used the *χ*^*2*^ tests and the u-tests to test for the difference in proportion and means among the three randomization groups. The success of randomization was evaluated in terms of the demographic variables collected in the baseline interview. The VarV coverage was compared among the two intervention groups and the control group (as reference), and the relative risk (*RR*) was calculated with the 95% confidence interval (*CI*). The VarV coverage was also compared with the DTP4 (as reference) among the three randomization groups, and the *RR* was calculated with the 95% *CI*. We used the *χ*^*2*^ tests to compare the difference in two intervention groups, with respect to the engagement of intervention activities, and the knowledge and attitude towards the VarV vaccination. A two-tailed *p*-value of < 0.05 was considered to be statistically significant. We used STATA MP 14.0 (Stata Corp. 2015, Stata statistical software, college station, TX, USA) for data analysis.

## Results

**Fig. 1:**
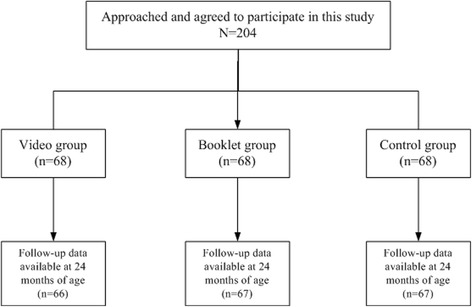
Participants enrollment of this study

A total of 204 pregnant women were approached for the recruitment. All of them agreed to participate in this study and completed the baseline questionnaires. Four children were lost to follow-up because they were not registered in ZJIIS and the mothers’ telephone numbers filled in the baseline questionnaires were incorrect and we could not contact them. Finally, two hundred pregnant women whose children’s VarV vaccination records could be verified through ZJIIS were included in our analysis (Fig. 1).

The average age of the 200 pregnant women were 25.8 years. The majority of respondents had a vocational or college education level (59.5%). 75.5% of them had a fixed job, and 61.5% of them were residents. The mean of gestational week at the time of enrollment was 16.8 weeks. The distributions of the demographics were not significantly different among the three randomization groups (Table 1).

**Table 1:** Demographic characteristics of the 200 pregnant women whose children’s vaccination status could be verified through ZJIIS

Demographics^a^	Total (*n* = 200)	Control group (*n* = 67)	Video group (*n* = 66)	Booklet group (*n* = 67)	*p*
Maternal age [years, mean(standard deviation)]	25.8 (3.5)	25.7 (4.1)	26.4 (3.4)	25.9 (3.5)	0.937
Maternal education level [*n* (%)]					0.995
≤ primary school	17 (8.5)	6 (9.0)	6 (9.1)	5(7.5)	
Middle school graduated	65 (32.5)	21(31.3)	22 (33.3)	22 (32.8)	
Vocational or college graduated	118 (59.0)	40 (59.7)	38 (57.6)	40 (59.7)	
Employment status [n (%)]					0.978
Employed	151 (75.5)	50 (74.6)	50 (75.8)	51 (76.1)	
Unemployed	49 (24.5)	17 (25.4)	16 (24.2)	16 (23.9)	
Immigration status [*n* (%)]					0.917
Resident	123 (61.5)	40 (59.7)	42 (63.6)	41 (61.2)	
Migrant	77 (38.5)	27 (40.3)	24 (36.4)	26 (38.8)	
Number of children [mean(standard deviation)]^b^	0 (0.3)	0 (0.4)	0 (0.3)	0 (0.2)	0.981
Gestational week at the time of enrollment [weeks, mean(standard deviation)]	16.8 (2.5)	16.6 (2.6)	16.7 (2.3)	17.0 (2.5)	0.992

^a^The difference in maternal age, number of children, and gestational age was tested through t-tests, while the difference in the other variables was tested through *χ*^*2*^ tests

^b^Not including the current pregnancy

In the control group, 56.7% of the children were vaccinated with VarV at the age of 24 months, including 38.8% vaccinated timely. In the video group, 86.4% of the children were vaccinated with VarV at the age of 24 months, including 70.1% vaccinated timely. In the booklet group, 76.1% of the children were vaccinated with VarV at the age of 24 months, including 61.2% vaccinated timely. The coverage of DTP4 by the age of 24 months was 95.2% for the control group, 94.8% for the video group, 95.7% for the booklet group.

In the video group, the *RR* (compared with control group) for VarV vaccination was 3.9 (95% *CI*: 1.9–8.0) at 12 months of age, and was 4.8 (95% *CI*: 2.1–11.3) at 24 months of age. In the booklet group, the *RR* (compared with control group) for VarV vaccination was 2.5 (95% *CI*: 1.2–5.0) at 12 months of age, and was 2.4 (95% *CI*: 1.2–5.1) at 24 months of age. The *RR* between VarV and DTP4 vaccination was 0.1 (95% *CI*: 0.02–0.2) in the control group, 0.3 (95% *CI*: 0.1–1.2) in the video group, and 0.2 (95% *CI*: 0.04–0.5) in the booklet group (Table 2).

**Table 2:** Comparison of the relative risk (RR) for children’s VarV and DTP vaccination

Group	12 months of age (n/N)	24 months of age (n/N)	DTP4 (n/N)	*RR*_-VarV/DTP4_^b^
Video	47/66	57/66	63/66	0.3 (95% *CI*:0.1–1.2)
Booklet	41/67	51/67	64/67	0.2 (95% *CI*:0.04–0.5)
Control	26/67	38/67	64/67	0.1 (95% *CI*:0.02–0.2)
*RR*_-video_^a^	3.9 (95% *CI*:1.9-8.0)	4.8 (95% *CI*:2.1–11.3)	–	–
*RR*_-booklet_^a^	2.5 (95% *CI*:1.2-5.0)	2.4 (95% *CI*:1.2–5.1)	–	–

^a^The reference for calculating the RR was the control group

^b^The reference for calculating the *RR* was the coverage of DTP4

Totally, there were 32 children receiving the delayed VarV vaccination within the time period of 13–24 months of age. The means (standard deviation) of delay were significantly different among the three groups (F = 10.6, *p* < 0.05), with 100.6(33.5) days in the control group, 57.3(11.2) days in the video group, 76.9 (8.2) days in the booklet group.

Engagement in the intervention activities, as assessed by the proportion of participants scored as “engaged” or “very engaged”, was significantly higher in the video group than in the booklet group (81.8% vs. 55.2%, *p* < 0.05). The proportions of women who felt they learned some knowledge on the VarV, and who believed in the information provided, and who clearly understood the education material did not significantly varied between the two intervention groups. The proportion of women who intended to vaccinate VarV for their children was significantly higher in the video group than the booklet group (93.9% vs 82.1%, *p <* 0.05).

## Discussion

To our knowledge, this was the first prospective randomized controlled trial to comparatively evaluate the effectiveness of the ELM-based health education interventions on the childhood VarV uptake in China. Also, research has been limited worldwide on the methods to improve the childhood VarV vaccination during mother’s pregnancy period.

Since the introduction of VarV, an increased coverage and a substantial decrease in the incidence of varicella disease had been observed in Zhejiang Province, but it was still lower than the VarV coverage in the USA, where the coverage was > 90% for children at the same age [5]. In this study, the VarV coverage of Changxing County didn’t meet the requirements for the varicella control and prevention due to the high basic reproduction rate of varicella [6]. Besides, a systematic review reported that approximately 15% of VarV recipients did not achieve the protective level of antibody and the immunity induced by VarV waned with time [7]. We assumed that a high incidence of varicella or even outbreaks in some population-dense settings, such as kindergartens or primary schools, would occur in Changxing due to its low coverage of VarV. However, we could not find the reasons for the low VarV coverage of Changxing in this study due to the limitation of the study design. We suggested that future researches should identify the areas with the low VarV coverage and investigate the specific determinants in different geographic areas or socio-economic strata.

Some previous studies indicated that decisions on receiving the childhood primary health service were based on the information presented to mothers [8, 9]. Similarly, the acquisition of sufficient knowledge and the better understanding are universally believed to be important determinants of successful immunization program [10–12]. Vaccination information can be delivered through a number of educational media, such as written pamphlets, videos, face-to-face counseling, and web-based applications [13]. However, when caregivers receive vast amounts of information at high speeds, it is often disconnected from meaning or purpose [14]. For example, nearly half of American adults have difficulty in comprehending health information, such as taking out of context or not fully understanding [15]. Specifically, caregivers with low health literacy may find it more challenging to comprehend and use health information on immunization effectively, mainly due to the complicated nature of the childhood immunization schedule [16, 17]. As such, health literacy on immunization should be addressed when developing interventions to facility caregivers to understand and use the information on immunization effectively. In this study, we compared the effects of the video and the booklet media. We found the VarV coverage among the two intervention groups was higher than that in the control group. Furthermore, the coverage in the video group was higher than that in the booklet group. These findings demonstrated that the two interventions had an optimal effect on improving the VarV coverage, and the video could be more effective as it might be more attractive. We assume that the use of video as an educational media offers several advantages. First, video can remove the potential inconsistencies across the educators and balance the presentation of information to provide more standardized education. Second, individuals with lower literacy especially prefer the video-based education as it can be easily understood, while the effectiveness of the written education materials such as booklet may be attenuated by the low literacy [18]. In fact, the video-based health education had been used to promote some specific preventive health behaviors and it significantly improved these behavioral outcomes [19–21]. Furthermore, the video intervention can be a less resource intensive way of delivering the educational information and can be administered in many forms, like the videotape, the digital video, the downloadable media files, and the streaming videos from Internet websites. These additional advantages would help the health education information spread quickly and reach a broad audiences via the social-media.

In this study, we found the coverage of VarV was much lower than that of DTP4 at the age of 24 months in both the control group and the booklet group. There may be several reasons for that. First, it may be explained that many health facilities would delay the voluntary vaccinations when the voluntary vaccines have to be administrated with other mandatory vaccines, according to the protocol that the mandatory vaccines have a higher priority [22]. Furthermore, simultaneous vaccination is still not widely practiced in Changxing county as the providers prefer to administrate one vaccine dose on the same clinic day, because they think it would be easier to identify the suspicious vaccine when the systemic adverse reactions (like fever) occur. Second, voluntary vaccines such as VarV need out-of-pocket fees and may be expensive sometimes, which would become a barrier for households with low income. Third, the difference in the coverage between DTP4 and VarV may resulted from the difference in the years of introduction of the vaccine [23]. As we know, DTP was introduced in 1978, the beginning of Chinese expanded program on immunization, while VarV was introduced in the late 1990s.

We found that the VarV coverage was similar to that of DTP4 among children in the video group by 24 months of age while it was not observed in the booklet group. We assume that it may be associated with the means of health education. Our previous study had identified that maternal education was positively associated with the vaccination coverage, including the voluntary vaccines [22]. The education level may influence the maternal knowledge and attitude and behavior on taking advantage of vaccination service for their children. Furthermore, the video-based health education may remove the literacy heterogeneity in the general population, and make all pregnant women understand the content of the education material easily.

Although the vaccination coverage is a critical measurement of the immunization program penetration, it may not be indicative of the vaccination timing. This study showed that the timeliness of VarV was significantly higher in the video group and the booklet group, compared with the control group. It appeared that the intervention groups with the affective messages on the VarV vaccination had a positive influence on the pregnant women to vaccinate their children with VarV at the recommended age, or on reducing the delay time of the VarV vaccination. Additionally, we found the timeliness of the VarV vaccination was higher in the video group than in the booklet group. We assumed that it would be associated with the different degrees of the engagement in these two intervention activities, which will be further discussed in the following part. As we know, delayed vaccination is an important concern of the public health. Delayed VarV vaccination can result in the accumulation of the susceptible persons to varicella. Furthermore, a delay in one vaccine can cause a domino effect inducing delays in the timeliness of other vaccine doses, which extends the period of the infective risk. Previous studies had indicated that untimely vaccination increased the failure of the completeness of immunization [24, 25]. Our findings emphasized the importance of the timeliness of VarV vaccination and suggested that the video-based health education should be an effective way to improve the vaccination administrated at the appropriate age.

The booklet developed in this study focused on the topics of the varicella and the VarV vaccination, but women in the video group were more engaged in the intervention activities and presented a better understanding of the education material. As a result, the video group had a higher proportion of intention to vaccinate their children with VarV. Previous researches on the use of entertainment health education had found mixed results on the effectiveness in completely behavior changing [26, 27]. However, of the two independent education interventions, the video was designed to evoke an emotional interaction with participants, through its affective entertainment education storyline, and it caused an improvement in the VarV coverage by both 12 and 24 months of age.

This study had several limitations. First, the enrolled pregnant women were limited to the population of a northwest county in Zhejiang province. Therefore, the results might not be generalizable to populations outside of the target geographic area. Second, we could not control the sample contamination as the pregnant women assigned into the control group might unintentionally read or watch the education materials on VarV vaccination and it would also very difficult to evaluate the potential influence on the results of this study. Third, the observed coverage in this study was higher than the assumptions made for the sample size calculation. The main reason was we used the results from the administration data on VarV coverage and the denominator might be inaccurate which underestimate the actual coverage of VarV. Fortunately, the current sample was still sufficient to detect the difference in the coverage of VarV at the actually higher values. Fourth, the recruitment procedure of this study was on a voluntary basis and it would potentially induce the selective bias, which would influence the accuracy of the results. Fifth, the questionnaire did not collect data on determinants from physicians or any other health-care workers, which would also be associated with the VarV vaccination.

## Conclusions

In this randomized controlled trial, the results indicated that perinatal health education through booklet or video could improve the coverage and the schedule adherence for childhood VarV vaccination. Our study also indicated that the health education on VarV vaccination in a video-based manner would be a better method to improve the VarV coverage. Further efforts need to focus on conducting larger studies among more the heterogeneous population.
